# Mucinous cystadenoma of the pancreas associated with pancreatic pseudocyst

**DOI:** 10.1093/jscr/rjad026

**Published:** 2023-02-06

**Authors:** Atsushi Horiuchi, Shun Akehi, Yousuke Abe, Nanako Ichikawa, Sakura Kawaharada, Sohei Kitazawa, Riko Kitazawa

**Affiliations:** Department of General Surgery, Ehime Prefectural Niihama Hospital, Niihama City, Ehime, Japan; Department of General Surgery, Ehime Prefectural Niihama Hospital, Niihama City, Ehime, Japan; Department of General Surgery, Ehime Prefectural Niihama Hospital, Niihama City, Ehime, Japan; Department of General Surgery, Ehime Prefectural Niihama Hospital, Niihama City, Ehime, Japan; Department of General Surgery, Ehime Prefectural Niihama Hospital, Niihama City, Ehime, Japan; Department of Molecular Pathology, Ehime University Graduate School of Medicine, Toon City, Ehime, Japan; Department of Molecular Pathology, Ehime University Graduate School of Medicine, Toon City, Ehime, Japan

## Abstract

Mucinous cystadenoma of the pancreas is considered as a premalignant lesion, and resection is recommended. The majority of pancreatic cystic lesions are pancreatic pseudocysts, so differentiation between mucinous cystadenoma and pseudocyst is frequently required. We report a rare case of mucinous cystadenoma of the pancreas coexisting with pseudocyst. A 43-year-old woman presented with abdominal pain. Imaging examinations showed a large cystic lesion in the tail of the pancreas, and distal pancreatectomy and splenectomy were performed. Pathological examination revealed that the majority of the cystic wall comprised thick collagen fibrous connective tissue, while part of the cystic wall represented a single layer of columnar, mucin-producing epithelium without atypia. Those findings suggested mucinous cystadenoma with an inflammatory pseudocyst. The mixture of mucinous cystadenoma and pseudocyst within the same cystic lesion appears to be very rare. Complete resection of the cystic lesion seems to allow an excellent prognosis.

## INTRODUCTION

Pancreatic mucinous cystic neoplasms (MCNs) are rare, mucin-producing, round cystic tumors with a fibrous capsule. MCNs are often seen among women in the 40- to 50-year-old age range and are most often located in the body and tail regions of the pancreas [1–3]. Reaching a diagnosis of MCN is easy if multiple cystic lesions with large amounts of mucus are present. However, differentiating MCN from pancreatic pseudocyst is difficult when the lesion is closer to a single cyst. Some reports have described characteristic views of MCN during observation under an initial diagnosis of pancreatic pseudocyst, with the true diagnosis of MCN made after resection of the cyst [4–6].

Few reports have described MCN associated with pancreatic pseudocyst [5, 7–10]. We describe herein a rare case of pancreatic mucinous cyst adenoma coexisting with inflammatory pseudocyst in the same cyst and discuss the case with reference to the literature.

## CASE REPORT

**Figure 1:**
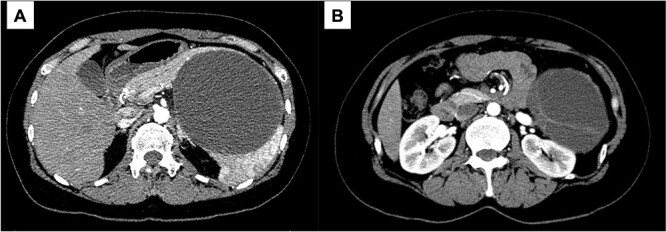
Contrast-enhanced CT; (**A**) the large cystic lesion in the pancreatic tail has displaced the stomach and the body of the pancreas; (**B**) the wall and septum show enhancement.

**Figure 2:**
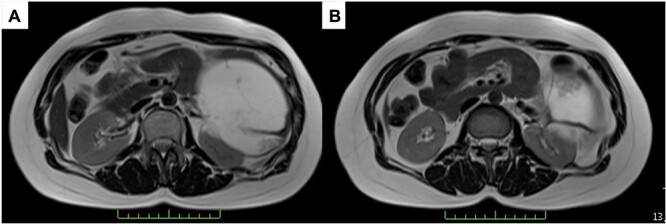
T2-weighted MRI; (**A**) the tumor has a thick fibrous capsule; (**B**) the tumor has a thick fibrous septum.

**Figure 3:**
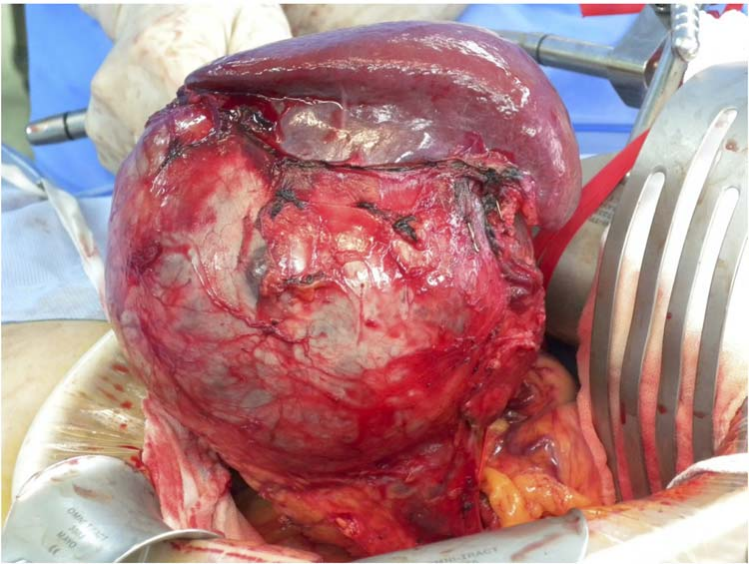
Intraoperative findings; a 15-cm tumor is identified in the tail of the pancreas, adherent to the spleen; distal pancreatectomy with splenectomy was performed.

A 43-year-old woman visited our hospital because of abdominal pain. Her medical history was unremarkable and not suggestive of acute pancreatitis or cholelithiasis. Laboratory studies revealed slight anemia and normal levels of amylase and inflammatory markers. Contrast-enhanced computed tomography (CT) showed a multilocular cystic lesion with a septum and a small, hyperdense area suggestive of intratumoral bleeding (Fig. 1). Magnetic resonance imaging (MRI) revealed a multilocular cystic lesion with a thick septum on T2-weighted imaging (Fig. 2). Those findings suggested a pancreatic cystic tumor, including the possibility of MCN, and we decided to perform distal pancreatectomy with splenectomy. A midline incision was made in the upper abdomen. A 15-cm tumor was identified in the tail of the pancreas, displacing the stomach and body of the pancreas. We mobilized the tumor in the tail of the pancreas along with the pancreatic body and spleen en bloc from the retroperitoneum. The tumor was extirpated by dissecting the body of the pancreas (Fig. 3).

**Figure 4:**
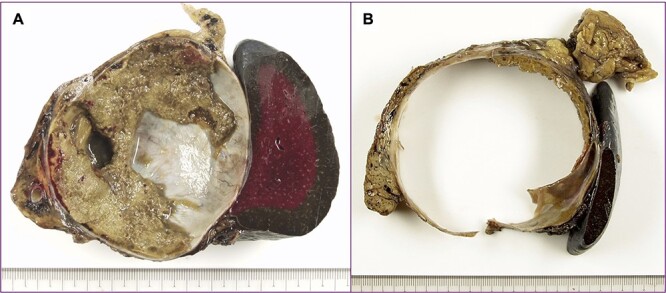
A cut surface of the tumor; (**A**) the tumor included necrosis tissue and septum; (**B**) the cystic wall was thick.

**Figure 5:**
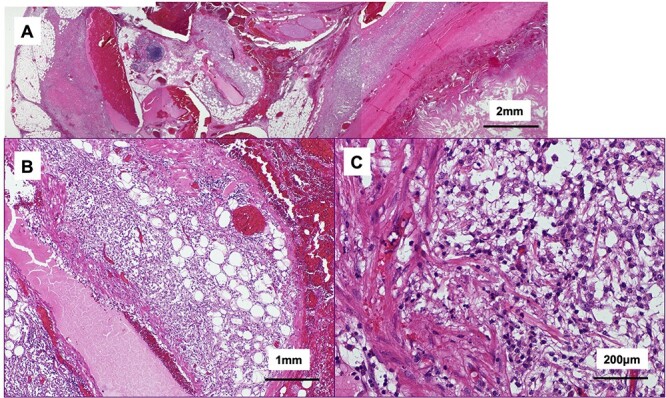
Histological findings of the tumor (hematoxylin and eosin stain); (**A**) the tumor included bled, necrosis tissue and cholesterin crystal; the cystic wall compromised collagenous, fibrous tissue; (**B**, **C**) eosinophilic necrosis and accumulated macrophages in fat outside the cystic wall.

**Figure 6:**
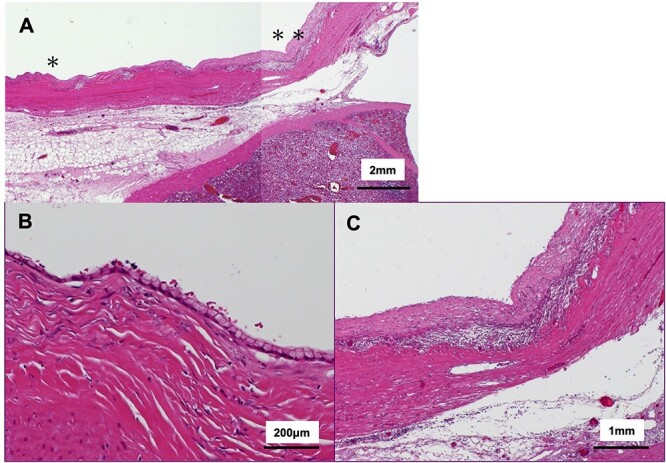
Histological findings of the cystic wall (hematoxylin and eosin stain); (**A**) ~95% of overlying cystic walls lacked apparent epithelium; (**B**) magnified view from ^*^ area in (A); ~5% of the cyst wall was covered with monolayered mucin-producing, goblet-like cells lacking high grade atypia; (**C**) magnified view from ^*^^*^ are in (A); ~95% of the cyst wall was covered with fibrous tissue surrounded by inflammatory cells.

The tumor included bled, necrosis tissue and cholesterin crystal (Figs 4 and 5) Pathological examination revealed that most of the cystic wall comprised collagenous, fibrous tissue without epithelium. The presence of eosinophilic necrosis and accumulated macrophages in fat tissues outside the cystic wall suggested inflammatory pseudocyst (Fig. 5) The cystic wall near the spleen included monolayer mucinous epithelium without cellular atypia. Based on those findings, mucinous cyst adenoma of the pancreas coexisting alongside pseudocyst was diagnosed (Fig. 6).

The post-operative period was complicated by Grade B pancreatic fistula and the patient was discharged 17 days after surgery. She has since been followed up for 8 years and has remained well without any signs of recurrence.

## DISCUSSION

Various types of pancreatic cystic lesions have been described, so differential diagnoses are very important because the treatment plans will vary depending on whether the lesion is neoplastic. Pancreatic pseudocyst is the most common, comprising 70–80% of pancreatic cystic lesions and occurring with acute pancreatitis [11–13]. Differentiation between neoplastic cysts and pseudocysts in the pancreas remains difficult and the misdiagnosis of pancreatic neoplasm as pseudocyst is not uncommon [14]. Although pancreatic pseudocyst is the most common cystic lesion of the pancreas, surprisingly, few cases associated with MCN have been reported [5, 7–10]. We have identified only six cases, including our own.

In all cases, the lesion was located in the tail of the pancreas, and adenoma was diagnosed. All cases were seen in women in the 40- to 50-year-old age range. In three of the six cases, cystic lesions increased in size during follow-up, or new cystic lesions were identified [5, 7, 10]. Eventually, those lesions were resected and MCN was diagnosed. For that reason, careful follow-up was considered to be necessary, although the clinical diagnosis was pancreatic pseudocyst. In one case, two cystic lesions were detected at the first medical examination. One cystic lesion in that case was a multilocular cyst diagnosed as MCN. The other cystic lesion was a unilocular cyst diagnosed as pancreatic pseudocyst [9]. In those four cases, MCN and pancreatic pseudocyst existed as separate lesions. MCN and pancreatic pseudocyst coexisting in the same cystic lesion was reported in only two cases, including our own [8].

The details remain unclear regarding whether pancreatic pseudocyst occasionally occurs with MCN or whether MCN involves factors that contribute to the creation of pancreatic pseudocyst. Yamashita *et al*. [5] reported a case with 10 years of follow-up in which the pancreatic cystic lesion initially decreased, but the cystic lesion was extracted because of an increase in the characteristic features of MCN. That report showed a new unilocular cystic lesion near the MCN, and pancreatic pseudocyst was diagnosed after resection. The authors thus supposed that the cyst wall ruptured due to increased internal pressure within the MCN and the pseudocyst appeared secondary to the retroperitoneal rupture of the MCN. Other report has described the retroperitoneal rupture of MCN that is associated with acute pancreatitis [15].

In the report such as the present case involving MCN and pseudocyst in a single cystic lesion, the authors supposed that the small focus of precancerous epithelium may have led to obstruction of the pancreatic duct, causing formation of a pseudocyst distal to the obstruction, because no risk factors for pancreatitis were identified in that case, such as heavy drinking or cholelithiasis [8]. In our case, we thought that the MCN was originally present and repeated inflammation within the MCN may have led to formation of a large cystic lesion because the patient had no antecedent history of pancreatitis and the cystic wall showed inflammatory thickening. However, the exact mechanisms by which MCN induced repeated inflammation and a pseudocyst remain unclear.

Among MCNs, the prognosis of adenoma is generally favorable [1–3]. Among the reports of MCN cases associated with pancreatic pseudocyst, long-term follow-up was only mentioned in one case, and that patient remained well 30 months after surgery [7]. Likewise, our case showed no sign of recurrence as of 8 years after surgery. If the associated pseudocyst originated from rupture of the MCN, recurrence, such as dissemination, remains a major concern. However, complete resection of the cystic lesion as in our case carries an expectation of favorable prognosis.

## Data Availability

The datasets obtained during this study are available from corresponding author on reasonable request.

## References

[1] Crippa S , Salvia R, Warshaw AL, Dominguez I, Bassi C, Falconi M, et al. Mucinous cystic neoplasm of the pancreas is not an aggressive entity. Ann Surg 2008;247:571–9. 18362619 10.1097/SLA.0b013e31811f4449 PMC3806104

[2] Yamao K , Yanagisawa A, Takahashi K, Kimura W, Doi R, Fukushima N, et al. Clinicopathological features and prognosis of mucinous cystic neoplasm with ovarian-type stroma. Pancreas 2011;40:67–71. 20924309 10.1097/MPA.0b013e3181f749d3

[3] Nilsson LN , Keane MG, Shamali A, Bocos JM, Van Zanten MM, Antila A, et al. Nature and management of pancreatic mucinous cystic neoplasm (MCN): a systematic review of the literature. Pancreatology 2016;16:1028–36. 27681503 10.1016/j.pan.2016.09.011

[4] Ishikawa T , Haruta J, Yamaguchi T, Doisaki M, Yama T, Murate K, et al. A case of mucinous cystic neoplasm of the pancreas misdiagnosed as a pancreatic pseudocyst at a initial exam and resected after a 2-year follow-up. J Med Ultrasonics 2015;42:257–65. 10.1007/s10396-014-0581-5 26576582

[5] Yamashita Y , Ito K, Noda Y, Kobayashi G, Obana T, Horaguchi J, et al. A case of mucinous cystadenoma of the pancreas resected after a 10-year follow-up. Nihon Shokakibyou Gakkai Zasshi 2011;108:1280–7. 21737981

[6] Shioyama E , Mitoro A, Ogawa H, Kubo T, Ozutumi T, Kitagawa K, et al. A pancreatic mucinous cystic neoplasm undergoing intriguing morphological changes over time and associated with recurrent pancreatitis. Medicine 2019;98:e16435. 31305468 10.1097/MD.0000000000016435 PMC6641744

[7] Sperti C , Pasquali C, Davol C, Pedrazzoli S. Mucinous cystadenoma of the pancreas as a cause of acute pancreatitis. Hepatogastroenterology 1998;45:2421–4. 9951936

[8] Fisher CP , Pope I, Garden OJ. Mucinous cystic tumour of the pancreas presenting with acute pancreatitis. HPB(Oxford) 2001;3:271–3. 18333030 10.1080/136518201753335782 PMC2020631

[9] Hsieh C-H , Tseng J-H, Huang S-F. Co-existence of a huge pseudocyst and mucinous cystadenoma: report of a case and the value of magnetic resonance imaging for differential diagnosis. Eur J Gastroenterol Hepatol 2002;14:191–4. 11981345 10.1097/00042737-200202000-00015

[10] Russell R , Sharp KW. Mucinous cystadenoma of the pancreas associated with acute pancreatitis and concurrent pancreatic pseudocyst. Am Surg 2005;71:292–7. 15943401

[11] Rabie ME , El Hakeem I, Al Shaini S, El Hadad A, Jamil S, Shah MT, et al. Pancreatic pseudocyst or a cystic tumor the pancreas? Chin J Cancer 2014;33:87–95. 23958054 10.5732/cjc.012.10296 PMC3935010

[12] Brugge WR . Diagnosis and management of cystic lesions of the pancreas. J Gastrointest Oncol 2015;6:375–88. 26261724 10.3978/j.issn.2078-6891.2015.057 PMC4502158

[13] Joshi U , Poudel P, Ghimire RK, Basnet B. Pancreatic pseudocyst or mucinous cystadenocarcinoma of pancreas? A diagnostic dilemma. Clin Case Rep 2017;5:501–4. 28396777 10.1002/ccr3.887 PMC5378858

[14] Ali S , Bashir A. Giant mucinous cystadenoma: case report with review of literature. Gland Surg 2014;3:207–10. 25207214 10.3978/j.issn.2227-684X.2014.03.01 PMC4139126

[15] Haddad A , Sebai A, Rhaiem R, Ghedira A, Makni A. Pancreatic mucinous cystadenoma doubly complicated by acute pancreatitis and retroperitoneal rupture. J Visc Surg 2019;156:72–4. 30197176 10.1016/j.jviscsurg.2018.08.011

